# Clinical Spectrum and Outcomes of Post-COVID Syndrome Among Adults in Rural Kerala: A Cohort Study

**DOI:** 10.7759/cureus.100434

**Published:** 2025-12-30

**Authors:** Veena K Puthenveedu, Sreelal T Prabhakaran, Aneesh Basheer

**Affiliations:** 1 Clinical and Epidemiological Research Centre, Dr. Moopen's Medical College, Wayanad, IND; 2 Community Medicine Department, Dr. Moopen's Medical College, Wayanad, IND; 3 General Medicine Department, SRM Medical College Hospital and Research Centre, SRM Institute of Science and Technology, Chennai, IND

**Keywords:** blood pressure, cohort study, covid-19 sequelae, exercise tolerance, long covid, post-covid syndrome, rural population, six-minute walk test

## Abstract

Background

Post-COVID syndrome (PCS) is a condition that affects some individuals who have recovered from COVID-19, characterized by persistent symptoms that last for weeks or months after the acute illness has resolved. Despite ongoing research on PCS, key gaps remain, including short follow-up, absence of control groups, small sample sizes, and limited representation of mild-to-moderate cases.

Objectives

The objective of this study is to determine the incidence proportion and outcomes of PCS over 12 months in a rural district of Kerala.

Methods

A prospective cohort study was conducted among 596 participants, of which 298 had a previous COVID-19 infection confirmed by reverse transcriptase polymerase chain reaction (RT-PCR) or antigen test (exposure group), while the remaining had no evidence of COVID-19 (control group). Field workers collected data by direct interviews. Study variables, including blood pressure (BP), blood sugar, oxygen (O_2_) saturation, and six-minute walk distance (6MWD), were recorded at six, nine, and 12 months following recruitment.

Results

The incidence proportion of PCS was 91.9% (95% CI: 88.8-95.0%) in the exposure cohort (i.e., 274/298) compared to 83.2% (95% CI: 79.0-87.4%) in the controls (i.e., 248/298) at six months. The differences in the incidence proportion of PCS between the groups were statistically significant. The differences in mean 6MWD at six, nine, and 12 months were statistically significant (p = 0.037), with a nadir at nine months. Mean systolic and diastolic BP were significantly higher in the exposure group than the control group at six and nine months, which converged by 12 months.

Conclusion

The persistence of symptoms and functional impairments was common among both the exposure and control groups, though more frequent in the exposure (COVID-19) group. The findings suggest that post-COVID morbidity may overlap with the background community symptom burden. Further, multicenter studies with serology and adjusted analyses are required to refine pathways for screening and long-term care.

## Introduction

The emergence of the novel coronavirus, SARS-CoV-2, in late 2019 precipitated an unprecedented global pandemic, leading to a multitude of clinical presentations in affected individuals. While substantial progress has been made in understanding the acute phase of COVID-19, attention is increasingly shifting towards the long-term consequences experienced by survivors of the disease.

Post-COVID syndrome (PCS) is a condition that affects some individuals who have recovered from COVID-19, characterized by persistent symptoms that last for weeks or months after the acute illness has resolved. According to a systematic review and meta-analysis, the prevalence of PCS varies widely across studies, ranging from 4% to 80% of individuals who have recovered from COVID-19 [1]. The symptoms of PCS can be diverse and may include fatigue, shortness of breath, chest pain, cough, palpitations, joint and muscle pain, headache, loss of taste or smell, gastrointestinal symptoms, skin rashes, depression, anxiety, and cognitive impairment, among others [1,2].

PCS lacks a universally accepted definition, leading to variability in estimated prevalence and symptom duration [1]. The World Health Organization (WHO) defines PCS as the continuation or development of new symptoms three months after the initial SARS-CoV-2 infection, with these symptoms lasting for at least two months with no other explanation [2]. The National Institute for Health and Care Excellence (NICE) in the UK defines PCS as signs and symptoms that develop during or following an infection consistent with COVID-19, continue for more than 12 weeks, and are not explained by an alternative diagnosis [3]. It can also occur in people who were never hospitalized for their initial COVID-19 infection [4].

Extensive research is ongoing in this important area; however, several lacunae still exist. Short follow-up, lack of control group, small sample sizes, and inadequate representation of mild to moderate severity disease that comprises the majority of post-COVID patients are major issues in the available literature.

This study aimed to assess the incidence proportion and persistence of PCS among individuals recovering from acute COVID-19 in a rural district of Kerala, over a period of 12 months, and to compare clinical and functional outcomes with a non-COVID control group.

## Materials and methods

Study design, setting, and data sources

A prospective cohort study was conducted in Wayanad district, in the northeastern region of Kerala, between June 2022 and October 2023. The recruitment was carried out between June and October 2022, followed by 12 months of follow-up, and each participant completed assessments at six, nine, and 12 months. During the COVID-19 pandemic, Dr. Moopen’s Medical College Hospital functioned as the designated tertiary care center for COVID-19 management in the district and received referrals from both government and private healthcare facilities across Wayanad. A line list of laboratory-confirmed COVID-19 cases was prepared using records available at this center and from the centralized lab portal after due permission from the government health authorities. Identified individuals were subsequently contacted and enrolled through community-based field visits. Additional eligible participants recovering from COVID-19 and controls were also identified from the same geographic areas during field visits.

Study population and sampling technique

The study population comprised an exposure and a control group. The exposure group participants were selected randomly from COVID-19-positive patients who were admitted at various COVID-19 treatment centers in Wayanad and from outpatients and home-isolated cases under the care areas of Wayanad district health and a tertiary care teaching hospital catering to this district after March 2021. A stratified random sampling proportionate to the population was employed to ensure representation of individuals of all taluks in Wayanad. The control group participants were selected from persons in the same catchment area who had no prior confirmed or suspected COVID-19 infection, verified through detailed baseline history and available test records. Controls included hospital attendees who tested negative by reverse transcriptase polymerase chain reaction (RT-PCR) or antigen testing, as well as healthy community members. To ensure accurate classification, participants with recent acute respiratory infections, uncontrolled chronic illnesses, or other conditions that could mimic post-viral symptoms were excluded. The individuals unable to comply with scheduled follow-up assessments were also excluded.

Sample size

Since PCS includes several symptoms and abnormalities, we used fatigue, which is the most common feature reported in most studies. Considering that fatigue is likely to be present in 15% of post-COVID patients (based on several studies) compared to 7% in the general population, assuming an alpha error of 5% and power of 80%, the minimum required sample size was calculated as 265 participants per group (total of 530) using the following formula:

$n = \frac{(Z_{\alpha/2} + Z_{\beta})^{2} \times \{ p_{1}(1 - p_{1}) + p_{2}(1 - p_{2}) \}}{(p_{1} - p_{2})^{2}}$

To allow for attrition, recruitment exceeded this minimum. The final analyzed sample comprised 596 participants (298 in each group), which is approximately 12.5% greater than the minimum required sample size, thereby strengthening the study’s statistical reliability.

Variables at baseline from the exposure cohort

We collected general demographic details, COVID-19 disease category at diagnosis, details of admission, condition at the time of discharge (in hospitalized participants), and COVID-19 vaccination status. Data regarding comorbidities (diabetes, hypertension, chronic lung disease, chronic heart disease, chronic kidney disease, chronic liver disease) were also collected. Prior history of confirmed COVID-19 infection was documented at baseline, and any new COVID-19 episodes during follow-up were assessed at each scheduled visit and recorded accordingly. 

Outcome variables

PCS was defined as per the most acceptable definition used currently - symptoms developing during or after COVID-19 infection and lasting beyond three months (12 weeks) from first symptom onset in the absence of an alternative etiology and lasting at least two months. Any persistent or new symptoms, readmissions, and deaths were determined to quantify PCS and other outcomes.

Data collection procedure

Data were collected by direct interviews using Epicollect5 (Centre for Genomic Pathogen Surveillance Team, Oxford, UK), in a predesigned format by trained field workers using tablets. Study variables were collected along with measurement of blood pressure (BP) (using digital BP apparatus), blood sugar (using glucometer), and oxygen saturation (O2) using pulse oximeter (resting and after six-minute walk test (6MWT) in those with normal resting saturation) by field workers. The first follow-up visit was conducted at six months after recruitment, with subsequent assessments at nine and 12 months.

Ethics consideration

Institutional Ethics Committee (IEC) approval was obtained prior to study initiation. Written informed consent was obtained from each participant. Confidentiality of data was ensured, and all data linked to participant identity was coded and stored in a password-protected database accessible only to the principal investigator. 

Data analysis

Data were entered in the Epicollect5 application and analyzed using the statistical software jamovi (version 2.6.45.0, Jonathon Love, Damian Dropmann, and Ravi Selker, Sydney, Australia). Continuous variables were summarized as means with standard deviation (SD), minimum and maximum values, while categorical variables were expressed as frequency (n) and percentage (%). The incidence proportion of PCS was calculated using the standard definition. Categorical variables were compared using the chi-square test. Changes in continuous variables over time within groups were analyzed using the repeated measures analysis of variance test (RM-ANOVA), and between-group comparisons were performed using an Independent t-test. Statistical significance was assessed using a two-tailed p-value of less than 0.05 (p < 0.05). 

## Results

**Figure 1:**
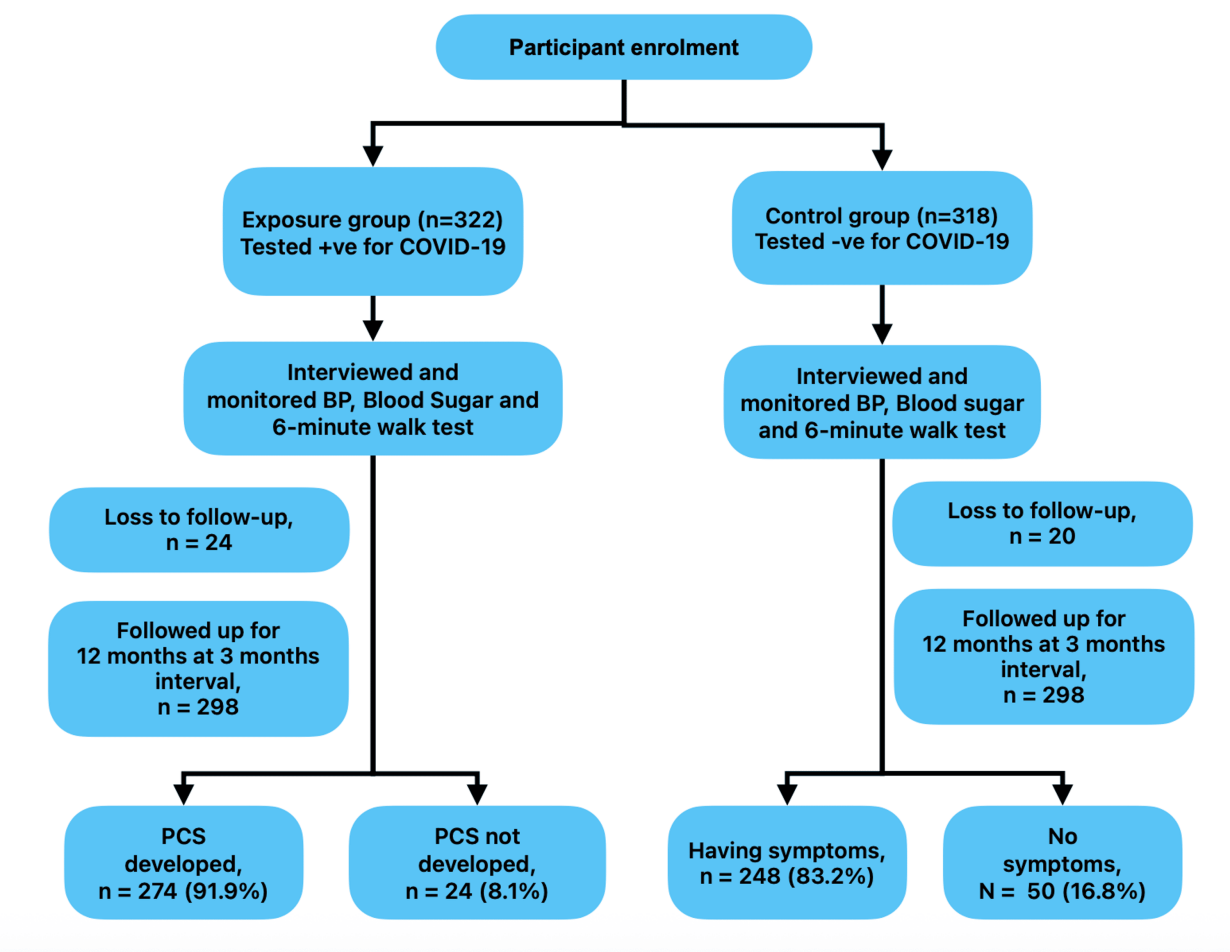
Flowchart of participant enrolment and follow-up PCS: post-COVID syndrome

A total of 640 participants were initially enrolled in the study, of which 322 belonged to the exposure group and 318 to the control group. After accounting for the loss to follow up (24 and 20, respectively), 596 participants were included in the final analysis. The details of participant enrolment and follow-up are shown in the flow diagram (Figure 1).

All 298 participants in the exposure group had a confirmed COVID-19 infection confirmed by RT-PCR or antigen test. Of these, 230 (77.2%) participants approached a private facility for testing. Within the exposure cohort, 172 (57.7%) participants underwent home-care isolation, whereas the remaining 126 participants required outpatient or inpatient care. Hospitalization was required only for 11 (3.7%) participants in the exposure cohort group, with a median hospital stay of 6 days (range: 1 to 17 days). The mean age of participants in the exposure group was 39.6 (11.7) years and 37.5 (12.3) years in the control group. The vaccine coverage was high in both groups, with 292 (98%) of the exposure group and 295 (99%) of the control group already vaccinated. The overall baseline characteristics of the participants are summarized in Table 1. 

**Table 1:** Baseline characteristics of participants in the exposure (n = 298) and control (n = 298) groups

Categories		Exposure group, n (%)	Control group, n (%)
Gender	Male	98 (32.9)	65 (21.8)
	Female	200 (67.1)	233 (78.2)
Vaccination status	Vaccinated	292 (98)	295 (99)
	Not vaccinated	6 (2)	3 (1)
No. of vaccine doses	0	6 (2)	3 (1)
	1	9 (3.1)	8 (2.7)
	2	224 (76.7)	176 (59.7)
	3	59 (20.2)	111 (37.6)
Comorbidities	Chronic kidney disease	0 (0.0)	1 (0.3)
	Chronic liver disease	2 (0.7)	0 (0.0)
	Chronic heart disease	5 (1.7)	4 (1.3)
	Hyperlipidemia	7 (2.3)	3 (1)
	Chronic lung disease	8 (2.7)	3 (1)
	Thyroid dysfunction	16 (5.4)	7 (2.3)
	Diabetes	20 (6.7)	14 (4.7)
	Hypertension	25 (8.4)	21 (7)
	Any of the above comorbidities	69 (23.2)	44 (14.8)

The clinical manifestations among the exposure group (n = 298) over a period of 12 months showed dynamic changes in the symptom prevalence (Table 2). Specifically, the participants with symptoms such as depressed mood increased from five (1.7%) at baseline to 10 (3.4%) at six months. The baseline to six months follow-up comparison showed that the frequency of participants with memory problems increased from 17 (5.7%) to 74 (24.8%), anxiety from 34 (11.4%) to 55 (18.5%), hair loss from 46 (15.4%) to 141 (47.3%) and those with shortness of breath from 116 (38.9%) to 158 (53%). In contrast, the symptoms such as loss of smell, loss of taste, headache, cough, myalgia, and fatigue showed a decrease in prevalence at six months compared to baseline (Table 2).

**Table 2:** Prevalence of clinical manifestations over a period of 12 months in the exposure group (n = 298)

Clinical features	Acute manifestation, (%)	Six months post-COVID, (%)	Nine months post-COVID, n (%)	12 months post-COVID, n (%)
Depressed mood	5 (1.7)	10 (3.4)	4 (1.3)	0 (0.0)
Loss of smell	91 (30.5)	37 (12.4)	1 (0.3)	0 (0.0)
Loss of taste	98 (32.9)	41 (13.8)	2 (0.6)	3 (1.0)
Anxiety	34 (11.4)	55 (18.5)	24 (8.1)	7 (2.3)
Reduced sleep	79 (26.5)	71 (23.8)	2 (0.6)	13 (4.4)
Memory problems	17 (5.7)	74 (24.8)	55 (18.5)	24 (8.1)
Headache	205 (68.8)	116 (38.9)	72 (24.2)	27 (9.1)
Hair loss	46 (15.4)	141 (47.3)	94 (34.3)	36 (12.1)
Cough	185 (62.1)	144 (48.3)	55 (18.5)	18 (6.0)
Myalgia	230 (77.2)	151 (50.7)	64 (21.5)	28 (9.4)
Shortness of breath	116 (38.9)	158 (53.0)	87 (29.2)	56 (18.8)
Fatigue	237 (79.5)	200 (67.11)	90 (30.2)	65 (21.8)

The follow-up analysis from baseline to 12 months among the exposure group showed a general decline in the prevalence of most symptoms. However, memory problems persisted at a higher number of participants, 24 (8.1%) at 12 months, compared to 17 (5.7%) at the baseline (Table 2).

Among the exposure group, only 24 (8.1%) participants regained their pre-COVID-19 health status at six months. Of the 298 participants in the exposure cohort, 274 (91.9%) had various symptoms (new or persistent) in the first six months after acute COVID-19 infection compared to 248 (83.2%) participants in the control cohort (χ² (1) = 10.43, p = 0.001). As per the definition, the proportion of PCS among the exposure group was 91.9% (274/298) (Table 3). At nine months, 12 (4.4%) of the exposure cohort required readmission for post-COVID-related problems compared to four (1.6%) of the control cohort (p = 0.051, Fisher's exact test).

**Table 3:** Presence of at least one symptom over a period of 12 months of follow-up in both groups (exposure group, n = 298; control group, n = 298) *Chi-square test, degree of freedom = 1

Follow-up period	Presence of at least one symptom	Exposure group, N = 298, n (%)	Control group, N = 298, n (%)	Test statistic	p-value*
Six months	Yes	274(91.9)	248(83.2)	χ² (1) = 10.43	0.001
Nine months	Yes	203(68.1)	138(46.3)	χ² (1) = 28.95	<0.001
12 months	Yes	127(42.6)	78(26.2)	χ² (1) = 17.85	<0.001

Persistence of at least one symptom at follow-up

At nine months of follow-up, 203 (68.1%) exposure group participants had at least one ongoing symptom, whereas only 138 (46.3%) control group participants had any of these symptoms (χ² (1) = 28.95, p <0.001). At 12 months of follow-up, 127 (42.6%) exposure group participants had ongoing symptoms compared to 78 (26.2%) control group participants (χ² (1) = 17.85, p < 0.001) (Table 3).

Number of symptoms over a period of 12 months

**Figure 2:**
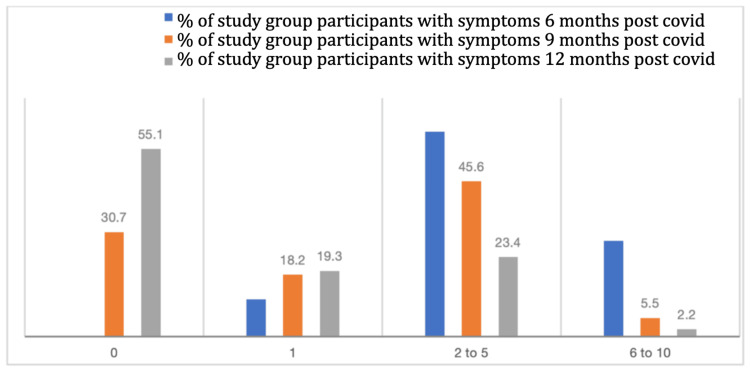
Distribution of number of symptoms over 12 months in the exposure group (n = 298) Categories displayed: 0, 1, 2 to 5, and 6 to 10 symptoms.

After six months of acute COVID-19 infection, 84 (28.1%) exposure cohort participants had six to 10 symptoms, compared to 27 (8.9%) control cohort participants. Additionally, two (0.7%) participants in the exposure cohort had more than 10 symptoms. These differences in the number of symptoms experienced by the exposure and control cohorts remained significant even at nine and 12 months of follow-up (Figure 2). Overall, fatigue was the most dominant and persistent symptom in the exposure cohort, reported by 237 (79.5%) during acute illness, 200 (67.1%) at six months, 90 (30.2%) at nine months, and 65 (21.8%) at 12 months. Systemic symptoms such as myalgia, headache, cough, and shortness of breath showed a steady decline over time, whereas neuro-psychological symptoms - particularly memory problems (5.7% at baseline to 24.8% at six months) and anxiety - were more prominent at six months and gradually decreased by 12 months. Hair loss showed a delayed peak at six months (47.3%) before declining.

Effect of COVID-19 on physical activity, blood pressure, blood sugar, and oxygen saturation

The comparison of cardio-metabolic parameters showed significant differences both between and within groups during the 12-month follow-up. The mean six-minute walk distance (6MWD) was 361.3 (115.9) m in the exposure group and 380.4 (93.9) m in the control group at six months (p = 0.03). This difference persisted between the groups at nine months (338.7 (131.9) m vs 357.6 (100.1) m; p = 0.05); however, at 12 months, the walk distance was similar in both groups (p = 0.98). The differences in 6MWD at different follow-ups (six, nine, and 12 months) were also statistically significant (p = 0.037), with the nadir reduction of walk distance coinciding with nine months post-infection (Tables 4-5).

**Table 4:** Comparison of mean differences in cardiometabolic parameters between exposure and control groups ^#^RM-ANOVA; ^*^independent t-test

Variables		Mean (SD)		Mean difference (95% CI)	p-value^#^	p-value*
		Exposure group	Control group			
6-minute walk distance (meters, m)	6 months	361.3 (115.9)	380.4 (93.9)	-19.1 (-36.1, -2.1)	0.063	0.03
	9 months	338.7 (131.9)	357.6 (100.1)	-18.9 (-37.7, -0.01)		0.05
	12 months	353.8 (122.8)	353.7 (111.5)	0.16 (-18.7, 19.0)		0.98
O_2_ saturation (%)	6 months	97.5 (1.8)	97.4 (5.3)	0.09 (-0.5, 0.7)	0.641	0.77
	9 months	97.3 (6.2)	99.9 (52.9)	-2.7 (-8.7, 3.4)		0.38
	12 months	97.7 (6.5)	96.6 (9.6)	1.1 (-0.3, 2.4)		0.12
Blood sugar (mg/dL)	6 months	120.9 (48.9)	117.6 (49.0)	3.4 (-4.5, 11.2)	0.126	0.4
	9 months	116.6 (50.5)	111.4 (40.3)	5.2 (-2.2, 12.5)		0.17
	12 months	126.9 (51.9)	120.01 (44.4)	6.9 (-0.8, 14.8)		0.08
Systolic blood pressure (mmHg)	6 months	123.7 (21.6)	119.8 (20.2)	3.9 (0.5, 7.2)	0.003	0.02
	9 months	123.3 (19.8)	117.1 (20.9)	6.1 (2.8, 9.4)		< 0.01
	12 months	122.7 (17.6)	120.1 (21.4)	2.5 (-0.6, 5.8)		0.12
Diastolic blood pressure (mmHg)	6 months	78.1 (16.0)	73.7 (13.4)	4.4 (2.1, 6.8)	0.001	<0.001
	9 months	77.1 (14.2)	73.7 (14.7)	3.4 (1.0, 5.7)		0.005
	12 months	75.17 (11.1)	74.1 (13.9)	1.1 (-0.9, 3.1)		0.296

**Table 5:** Comparison of mean differences of cardiometabolic parameters over time in the exposure group (n = 298) ^#^RM-ANOVA

Variables		Mean (SD)	p-value^#^
		Exposure group	
Six-minute walk distance (meters, m)	6 months	361.3 (115.9)	0.037
	9 months	338.7 (131.9)	
	12 months	353.8 (122.8)	
O_2_ saturation (%)	6 months	97.5 (1.8)	0.637
	9 months	97.3 (6.2)	
	12 months	97.7 (6.5)	
Blood sugar (mg/dL)	6 months	120.9 (48.9)	<0.001
	9 months	116.6 (50.5)	
	12 months	126.9 (51.9)	
Systolic blood pressure (mmHg)	6 months	123.7 (21.6)	0.642
	9 months	123.3 (19.8)	
	12 months	122.7 (17.6)	
Diastolic blood pressure (mmHg)	6 months	78.1 (16.0)	0.005
	9 months	77.1 (14.2)	
	12 months	75.17 (11.1)	

The mean systolic BP was significantly higher in the exposure group compared to the control group at both six and nine months follow-up (p = 0.02 and p = 0.01, respectively). Similarly, the exposure group had an elevated diastolic BP at six and nine months compared to the control group, which was statistically significant (p < 0.001 and p = 0.005, respectively). However, this difference was not evident by 12 months (Table 4). 

Within-group analysis in the exposure group showed a gradual decrease in the mean diastolic BP over a period of 12 months (p = 0.005). Mean blood sugar level showed a significant temporal variation (p < 0.001), although between-group differences were not statistically significant. Oxygen saturation did not show any significant variation during follow-up, either within or between group comparisons (Tables 4-5).

## Discussion

This prospective cohort study on the clinical manifestations, risk factors, and outcomes of PCS over 12 months in a rural district of Kerala identified that nearly 92% (95% CI 88.8-95.0%) of those who recovered from acute infection suffered from PCS. This proportion was significantly higher than that of 41.7% (95% CI: 39.7-43.8%) reported in a meta-analysis of 48 studies [5]. This high proportion of symptoms among the control group may suggest high background rates, due to undetected COVID-19, effects of vaccination, or other psycho-social factors. The comparative analysis shows higher comorbidity and vaccination rates among the exposure group. While fatigue remained the most common post-COVID symptom, its frequency reduced over a period of 12 months from acute infection. COVID-19 resulted in considerable long-term cardiorespiratory morbidity as evidenced by statistically significant reductions in 6MWD and increases in systolic BP.

Unlike other studies conducted in Kerala, this study followed up participants for a longer period of 12 months [6,7]. In our study, 96.3% of the study group participants were affected with mild or moderate acute COVID-19 infection (maybe because of the omicron wave). However, the study is relevant in the sense that even though the COVID-19 infection was mild or moderate, post-COVID morbidity appears to last for months. Incidence of some of the symptoms after six months, nine months, and 12 months following acute COVID-19 infection is a significant finding. 91.9% of exposure group patients had at least one symptom six months post-COVID compared to controls. Among the exposure group patients, 68.1% had persistence of at least one symptom after nine months, and 42.6% had ongoing symptoms even after 12 months. Studies from other parts of India, including northern states, have also documented post-COVID sequelae; however, most had shorter follow-up durations (typically three to six months) and were largely hospital-based without a concurrent control group, limiting direct comparability [8,9]. In contrast, the present study’s 12-month follow-up with a control cohort provides additional insight into the temporal evolution and persistence of post-COVID symptoms in a rural Indian setting.

Like many other studies, a predominance of neurological symptoms was observed in the present study, with a notable increase in prevalence after six months following acute COVID-19 infection. Evidence from studies including patients with asymptomatic or mild disease suggests that these manifestations may be mediated by brain-directed autoimmune processes and associated neural injury [10]. The evidence reveals that COVID-19 infection can initiate brain autoimmunity by activating microglia and triggering chronic neuroinflammation [11], producing autoantibodies against brain proteins [12], and causing blood-brain barrier impairment [13]. Specifically, patients demonstrate elevated brain injury markers (neurofilament light and glial fibrillary acidic protein) associated with autoantibody presence and pro-inflammatory cytokines [12]. Patients can develop multiple autoantibodies, with some experiencing neurological sequelae like anosmia and potential autoimmune disease onset [14]. The mechanisms include molecular mimicry, bystander activation, and epitope spreading, suggesting a robust and multifaceted autoimmune response to COVID-19 infection. Loss of smell and taste was noted in more than 10% of post-COVID patients even after six months of infection, and in lesser proportions after nine months of infection. Readmission percentage was low as the majority of the patients suffered from mild or moderate COVID-19 infection. The pattern observed in this cohort-early predominance of systemic symptoms followed by persistence of neuro-psychological and functional complaints-aligns with mechanisms proposed in recent post-COVID research. Prolonged fatigue, myalgia, and dyspnea may reflect ongoing immune dysregulation and post-viral inflammatory changes. Studies have demonstrated persistent cytokine activation, autonomic dysfunction, and the presence of autoantibodies that may contribute to “brain fog” and delayed recovery. The delayed peak in hair loss corresponds to telogen effluvium, a well-recognized sequela of systemic illness or stress. These findings parallel the trajectories reported in large cohort studies and systematic reviews, underscoring the multifactorial pathophysiology underlying PCS [1,10,15,16].

A study in the US found that 31% of outpatients with COVID-19 reported symptoms lasting beyond six months [10]. Another study conducted in Italy found that 87% of patients hospitalized with COVID-19 had at least one persistent symptom two months after discharge [15]. A study from the UK reported that 10% of patients with mild COVID-19 infection experienced symptoms lasting beyond 12 weeks [16]. However, in our study, a significant proportion of exposure group patients reported persistence of more than six symptoms after six months of COVID infection. After nine months post-COVID, about 6% patients had six to 10 symptoms that affected their daily activities, while around 2.5% patients had six to 10 symptoms even after one year. 

Our study found an interesting trajectory of cardiometabolic parameters in post-COVID patients. At six months of follow-up, the 6MWD was significantly lower among post-COVID patients compared to controls. Moreover, this distance dropped further at nine months while still showing a statistically significant difference with controls. Interestingly, by 12 months, 6MWD increased and was no longer different from controls. This indicates that the exercise tolerance of post-COVID patients is reduced with maximum impairment at nine months post-infection, despite the majority of the exposure group participants having mild to moderate disease, not requiring hospitalization. It must also be noted that there is a trend toward recovery or improvement at around one year. A longer follow-up of these patients might provide insights as to whether there is complete recovery or return to baseline exercise tolerance. The 6MWD has been used in many conditions as a marker of disease severity and to monitor progression [17,18]. Its use in COVID-19 and PCS has been limited despite its potential as a simple and effective marker of the severity of acute disease and progression of chronic effects. The 6MWD at six months in post-COVID patients correlated positively with DLCO and negatively with the HRCT severity score [19]. A prospective study on 85 patients from Taiwan who were discharged from the hospital after COVID-19 found that the mean 6MWD was shorter in those with severe pneumonia at 60 days, although it was not statistically different from those with mild disease and non-severe pneumonia [20]. A study reported an improvement in the 6MWD among post-COVID patients followed up at three months compared to one month. However, this study included only patients with dyspnea [21]. Some studies followed up patients for longer periods of time to assess the respiratory functions, including the six-minute walk test; however, they included only patients who recovered from COVID-19 pneumonia, limiting the generalizability to the large proportion with mild disease [22]. Wong and colleagues also demonstrated a significant reduction in the 6MWD among patients who recovered from severe COVID-19 compared to those with mild disease [23], but unlike our study, there was no further follow-up of these patients to determine trends over time.

A similar trend was noted for changes in systolic and diastolic BPs as well. Post-COVID patients had higher systolic and diastolic BP compared to controls at six and nine months follow-up. The absolute mean differences between the exposure and control groups in systolic BP were around 5 mmHg and 10 mmHg at six and nine months, respectively. Again, like the six-minute walk test, the BP returned to near baseline levels by 12 months with no significant difference from controls. Further, despite such increases, the mean systolic BP and diastolic remained below 130 mmHg and 80 mmHg, respectively, till the end of the study. Therefore, the clinical significance of the elevations in BP among post-COVID patients compared to controls remains elusive. A systematic review on cardiovascular sequelae in PCS reported significantly higher risk for hypertension in these individuals compared to unaffected persons with an Odds ratio of 1.65 (95% CI 1.55-1.77). However, the absolute changes in BPs were not discussed in the meta-analysis [24]. Most studies included in the review were retrospective or case-control studies. The few prospective studies in the review had follow-up periods ranging from three to six months. 

The major strength of this study is the inclusion of a control cohort, which was also followed up for the same duration as the exposure group. Further, the large sample size and minimal loss to follow-up add considerable internal validity. To the best of our knowledge, this is one of the very few studies from India on PCS that compared exposure and control groups and obtained data through direct interviews and regular follow-ups for one year. Besides, the majority of patients in the exposure group were out-of-hospital, closely resembling the real-world COVID-19 situation, making our results more generalizable [25].

While every effort was made to ensure the accuracy and reliability of findings, it is crucial to acknowledge some limitations of this study. We did not employ any diagnostic testing to confirm the COVID-19 status of control group participants, relying instead on self-reported symptoms and medical history. This reliance on self-reporting introduces the possibility of misclassification and underreporting of COVID-19 cases, potentially leading to an inaccurate representation of the infection status within the recruited control group. Consequently, the lack of laboratory-confirmed testing may have impacted the precision of the study's outcomes, as undetected COVID-19 cases among control group participants could have influenced the observed results. Future research should consider incorporating rigorous diagnostic testing protocols to enhance the reliability of participant recruitment and ensure a more accurate delineation of COVID-19 status among study participants.

## Conclusions

In this prospective cohort from rural Kerala, a substantial proportion of the individuals who recovered from acute COVID-19 disease reported more persistent symptoms and functional impairments compared to the control group. However, it was observed that the background symptom prevalence was also high. Although controls reported a high background symptom burden, the post-COVID patients showed greater symptoms and functional limitations until convergence over time, with a subset remaining symptomatic at one year. While these findings support targeted follow-up between three and nine months after infection, the overlap with the community-level symptoms indicates that PCS should be interpreted within a broader biopsychosocial framework. Future multicentric studies incorporating serology, functional testing, and adjustment for vaccination, comorbidities, and psychosocial stressors are needed to refine screening and long-term care pathways.
